# Early Seedling Screening Reveals Unidentified Al Resistance Mechanisms in Lithuanian Barley Cultivars

**DOI:** 10.3390/ijms26083803

**Published:** 2025-04-17

**Authors:** Vilius Jurgis Mensonas, Violeta Kleizaitė, Algė Leistrumaitė, Raimondas Šiukšta

**Affiliations:** 1 Institute of Biosciences, Life Sciences Center, Vilnius University, Saulėtekis Ave. 7, 10257 Vilnius, Lithuania; vilius.mensonas@gmc.vu.lt (V.J.M.);; 2 Institute of Agriculture, Lithuanian Research Centre for Agriculture and Forestry, Institutas Ave. 1, Akademija, 58344 Kėdainiai, Lithuania

**Keywords:** aluminum resistance, aquaporins, barley, biochemical markers, *HvAACT1* expression, *TIP4;1*

## Abstract

Aluminum toxicity in acidic soils represents a significant environmental stressor that affects yields worldwide and is only expected to worsen. Breeding resistant varieties remains the most viable solution; however, fast and robust procedures to determine cultivar viability must be developed and applied to promising genotypes. This study explored historical and modern Lithuanian-bred barley cultivars using morphometrical and biochemical markers for Al resistance and sequence and expression analyses of potential candidate genes. Morphometric seedling measurements (relative root length reduction −13.65 ± 0.33% (*p* < 0.001) and root tolerance index 0.86 ± 0.44 after 72 h at 8 mM Al stress) revealed the modern cv. ‘Ema DS’ to be the most Al resistant, while biochemical assays offered a poor distinction between the Al-resistant and sensitive cultivars. Thus, we determined that morphometric parameters were more effective in the early screening for barley Al resistance. The genetic screening of well-established Al resistance markers in the barley citrate transporter HvAACT1 revealed a mismatch between the observed barley phenotypes and genotypes. Further testing was conducted through expression analyses of *HvAACT1* and seven aquaporin family genes, which revealed a correlation between the best empirical performance in cv. ‘Ema DS’ and a high *HvAACT1* (2.02 fold change, *p* < 0.05) expression, despite the lack of established genetic markers, as well as a stress-induced significant upregulation of aquaporin TIP4;1 (2.45 fold change, *p* < 0.05), suggesting previously undiscovered regulatory mechanisms of external and internal detoxification influencing Al resistance in Lithuanian barley cultivars, as well as potential future candidates for Al-resistant barley breeding programs.

## 1. Introduction

With evidence of cultivation dating back almost 10,000 years, barley (*Hordeum vulgare* L.) is one of the earliest domesticated crops and has remained an agricultural mainstay ever since [1]. Despite the gradual decline in its role as a primary food source due to the rise of wheat and other cereal crops, barley currently ranks as the fourth most important cereal in the world, behind wheat, corn, and rice [2]. Mostly used as livestock feed, in malting and brewing, and recently in biofuel production, barley crop farming has remained a major economic contributor to many countries with developed agricultural sectors [3].

However, despite having a reputation as a particularly adaptable cereal crop with many seasonal cultivars and numerous genetic adaptations, enabling it to grow in a variety of climates and areas [1], barley is nevertheless intensely affected by various abiotic environmental stresses [4,5].

Soil acidity ranks among the most important of these environmental stresses, affecting 30–70% of the world’s arable land [6,7,8] and resulting in damage of billions of dollars [9]. It is a major plant growth limiting factor, reducing the availability of soil nutrients, limiting water uptake, and disrupting the balance of elements within the soil [8]. Unsurprisingly, all stages of plant development are affected by lower environmental pH values, from a reduced seed germination rate, inhibited vegetative growth, and a reduced photosynthetic efficiency to a reduced reproductive capacity and lower yield [10]. However, while a decrease in the pH itself affects a variety of plant metabolic and cellular processes [11], soil acidity also increases the solubility of many toxic metal ions, of which Al ions are among the most damaging [8].

Increased concentrations of soluble Al ions (Al^3+^) are considered the main reason for poor plant growth in acidic soils [10,12]. In low-pH environments, various Al compounds, such as Al oxides, sulfates, and silicates, dissolve and release free Al ions, which cause a variety of harmful effects, such as the inhibition of root growth, an increase in the root cell wall rigidity, and the inhibition of DNA replication [10].

To combat the adverse effects of Al poisoning, plants have developed a wide variety of mechanisms. These mechanisms are generally divided into two main groups—mechanisms of Al exclusion and mechanisms of internal tolerance [13]. The internal mechanisms of Al tolerance rely on the intracellular chelation of Al ions, coupled with vacuolar isolation and translocation to less Al-sensitive organs in the plant. In addition to several other processes, such as reducing the local rhizosphere pH or manipulating the cell wall and membrane characteristics to limit Al uptake, the external mechanisms of Al exclusion function similarly to internal tolerance [12]. Organic acids, such as oxalate, malate, or citrate, are exuded from the plant root tips and chelate Al^3+^ ions to create non-toxic compounds that do not damage the roots [13]. This external mechanism of Al poisoning resistance is universal to many cereal crops, as well as other plants [12,14]. Barley crops are no exception, and the chief mechanism of Al resistance in barley is the external secretion of citrate in response to increased Al^3+^ concentrations [15].

A great deal of progress has been made throughout the last few decades in terms of describing the physiological processes of Al resistance, as well as their genetic basis, such as the identification of the gene controlling citrate secretion in barley roots, which is named *HvAACT1* [16]. Further research has revealed polymorphisms in the UTR regions of the *HvAACT1* gene, as well as SNPs in its coding sequence, all of which contribute to the phenotype of Al-resistant barley cultivars [16,17,18,19]. However, many questions remain unsolved, as the interplay between various genetic factors contributing to barley Al resistance is still unclear, and even the best-described mechanisms account for only part of the phenotypic variation observed between barley cultivars [18,19].

In Lithuania, soil acidity was first measured on a country-wide scale during the 1960s and was found to be a major issue, with almost half—approximately 40%—of soils measuring below the cutoff pH considered acidic. During the 1970s and 1980s, a large state-sponsored liming program was introduced with the aim of reducing the acidity of arable soils in Lithuania, which was quite successful, and the total acidic soil area in Lithuania was reduced to just 18.6% in 1991 [20]. However, after the restoration of Lithuanian independence, this program was discontinued, and soil liming was left to the discretion of individual farmers. Since then, acidic soils have gradually returned to Lithuania, with approximately 27% in 2023 [20,21].

The genetic diversity of many edible crops has dramatically decreased due to bottlenecks during domestication and subsequent modern breeding. The most heterogeneous populations of barley were grown until the late 19th century, after which the genetic diversity declined consistently, making barley wild genotypes and historical landraces the most promising sources of diversity [22,23,24,25]. Lithuanian-bred barley cultivars are no exception, with modern cultivars following the more rigorous standards of the DUS (distinctness, uniformity, and stability) system [26]. This offers a unique opportunity to study the genetic resources and look into potential Al resistance mechanisms of Lithuanian barley cultivars, of which the historical genotypes were developed before the state-wide liming efforts and rigorous breeding standards, whereas newer cultivars emerged after the end of the program and the implementation of strict breeding criteria. Neither group has ever been assayed for soil acidity resistance nor genetic mechanisms thereof, providing an excellent opportunity for this study to evaluate and compare the early Al-resistance phenotypes of heterogeneous local historical cultivars, suspected Al-resistant cultivars, and international Al-resistant genotypes using standard morphometric, biochemical, and molecular biomarkers. This analysis allowed us to determine the most efficient Al resistance screening biomarkers at early seedling stages, as well as to identify Al-resistant local cultivars and potential new internal Al resistance mechanisms in barley.

## 2. Results

### 2.1. Evaluation of Al Resistance Using Morphometric Parameters

The root and shoot lengths of the barley seedlings were measured before exposure to the stress conditions, as well as at 24-h intervals post-transfer (24, 48, and 72 h). Notably, after 24 h, significant differences in the shoot length sizes were observed in the ‘Rusnė DS’ cultivar, in which the shoot lengths in both the 2 mM Al and 8 mM Al stress groups were significantly (*p* < 0.001) shorter (−16.89 ± 0.22 and −12.29 ± 0.18, respectively) than those in the control group, as was the case for cultivar ‘Auksiniai 3’, in which only the 8 mM group (−13.79 ± 0.16) presented a significant reduction (*p* < 0.05, Table 1). At 48 h post-transfer, the shoot growth was significantly inhibited in the ‘Alisa DS’, ‘Arka DS’, ‘Ema DS’, ‘Noja DS’, and ‘Rusnė DS’ cultivars. Among the historical cultivars, only cv. ‘Auksiniai 3’ and cv. ‘Ūla’ performed significantly worse than the control, both in the 8 mM groups. By the 72-h time point, shoot growth differences were observed in the majority of cultivars, where cv. ‘Bavaria’ and cv. ‘Kirsna DS’ among the modern cultivars, as well as cv. ‘Alsa’, cv. ‘Auksiniai II’, and cv. ‘Džiugiai’ among the historical cultivars, exhibited insignificant reductions in the shoot length.

In terms of the root length, at the 24-h mark, significant differences were evident in the ‘Alisa DS’, ‘Arka DS’, ‘Noja DS’, ‘Kirsna DS’, and ‘Rusnė DS’ cultivars, as well as cv. ‘Aidas’ and cv. ‘Auksiniai 3’, where one or both stress groups exhibited reduced growth rates (Table 2). At 48 h post-transfer, the root growth had significantly decreased across all the modern cultivars, whereas among the historical cultivars, only cv. ‘Aidas’, cv. ‘Auksiniai 3’, and cv. ‘Ūla’ presented significant reductions. The root growth inhibition was also apparent at 72 h for all the tested modern cultivars, with significant differences discernible between the two stress environments in the ‘Alisa DS’, ‘Bavaria’, ‘Kirsna DS’, and ‘Noja DS’ cultivars. Among the historical cultivars, only cv. ‘Alsa’ and cv. ‘Džiugiai’ presented no significant reductions in the root length in either stress group.

An analysis of the results revealed that the root response displayed greater uniformity and predictability across all the tested cultivars. The lowest response was observed on the first day, which subsequently increased with increasing duration in the stressful environment. The trend of the increasing response was not as strong among the historical cultivars, where some cultivars, such as cv. ‘Alsa’ or cv. ‘Džiugiai’, did not produce significant decreases in the root length even after 72 h in the stressful environment. Conversely, the response strength of the leaves demonstrated more substantial variation: some cultivars presented a progressive increase in response strength, whereas others presented the greatest response on day 1, followed by a decline over time. Notably, among the historical cultivars, cv. ‘Džiugiai’ displayed a response that peaked on day 1 in both stress groups and later increased to positive levels. Nonetheless, these changes were not statistically significant.

To elucidate the trends among cultivars on the basis of morphological indicators, two additional derivative parameters were employed, RTI and the RRLR/ABS RL correlation, which were calculated during the seedling growth period and before the biochemical assays.

**Figure 1:**
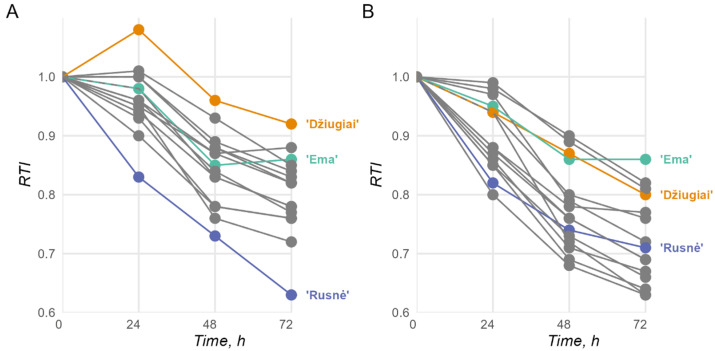
Barley seedling root tolerance index (RTI) measurements over 72 h in 2 mM (**A**) and 8 mM (**B**) Al stress environments (*n* > 30 for each timepoint). The lines indicate assayed cultivars, as described in the Materials and Methods. The most relevant cultivars are highlighted, while other cultivars are presented in grey; the full data with the remaining observations can be found in Table S1.

RTI measurements, expressed numerically, range from 0 to 1, with 1 representing the highest level of tolerance; over 72 h, these measurements presented the expected trends; all cultivars, excluding cv. ‘Rusnė DS’, presented a lower RTI in the 8 mM Al stress group. In the 2 mM stress groups, the most tolerant cultivars were identified as ‘Bavaria’, ‘Ema DS’, and ‘Kirsna DS’ among the modern cultivars and as ‘Džiugiai’, ‘Auksiniai II’, ‘Auksiniai 3’, ‘Ūla’, and ‘Luokė’ among the historical cultivars (Figure 1A). Overall, cv. ‘Džiugiai’ was the most tolerant of all the cultivars, and cv. ‘Ema DS’ was the standout cultivar among the modern cultivars, especially with increasing RTIs during the 48–72 h period, possibly indicating adaptation to the stressful conditions. In the 8 mM group, the standard Al-resistant cultivar ‘Bavaria’ lost its advantage, which left cv. ‘Ema DS’ as the standout cultivar in terms of Al resistance (Figure 1B). A decline in the RTI was also noticeable among the historical cultivars, where only cv. ‘Auksiniai II’, cv. ‘Luokė’, and cv. ‘Džiugiai’ remained competitive with cv. ‘Ema DS’. Notably, the decline in tolerance between the 2 mM and 8 mM groups for cv. ‘Ema DS’ was negligible at most. The full RTI measurements can be found in Table S1.

**Figure 2:**
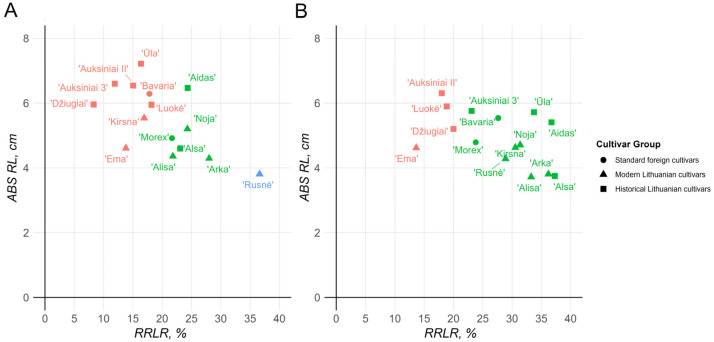
Barley seedling relative root length reduction (RRLR)/absolute root length (ABS RL) relationships after 72 h exposure to 2 mM (**A**) and 8 mM (**B**) Al stress (*n* > 30). Different colors depict the different sensitivities of the cultivars to Al stress: red represents tolerant, green represents moderately tolerant, and blue represents sensitive barley genotypes.

RRLR/ABS RL correlation calculations confirmed previously observed patterns. In the 2 mM Al stress environment, three distinct groups of cultivars could be observed, where ‘Bavaria’, ‘Ema DS’, ‘Kirsna DS’, ‘Džiugiai’, ‘Auksiniai II’, ‘Auksiniai 3’, ‘Ūla’, and ‘Luokė’ identified as the most resistant cultivars in the RTI calculations made up one of the clusters, and the remaining cultivars made up the second cluster (Figure 2A). One standout among these groups was cv. ‘Rusnė DS’, which exhibited such an extreme reduction in root length that it could not be clustered with either of the major groups. Similarly, in the 8 mM environment, two major groups could be identified; however, there was significantly less distinction between them (Figure 2B). The first group consisted of cv. ‘Ema DS’, cv. ‘Auksiniai II’, cv. ‘Luokė’, and cv. ‘Džiugiai’ and the rest of the remaining cultivars. Notably, cv. ‘Rusnė DS’ performed better under the 8 mM stress conditions than under the 2 mM stress conditions, indicating a major decrease in performance under harsher conditions among the other cultivars.

Compared with the standout Lithuanian candidate cv. ‘Ema DS’, in the 2 mM stress group, the majority of historical cultivars presented greater reductions in the root length, except for cv. ‘Džiugiai’ and cv. ‘Auksiniai 3’, which growths were inhibited by approximately 5% and 2%, respectively. In the 8 mM environment, however, cv. ‘Ema DS’ remained the most resistant cultivar, with an approximately 14% reduction in root length, whereas both cv. ‘Džiugiai’ and cv. ‘Auksiniai 3’ showed approximately 19% and 23% reductions, respectively.

### 2.2. Evaluation of Al Resistance Using Biochemical Parameters

At the 72-h time point after the seedlings were transferred to the stressful environments, root and shoot samples were collected and assessed for lipid peroxidation levels, chlorophyll and carotenoid contents, Al contents, and cell viability.

Most of the observed parameters did not significantly differ between the same cultivar stress and control groups or between the respective standard cv. ‘Bavaria’ groups. Some notable exceptions were the total chlorophyll level measurements, for which most modern Lithuanian cultivars presented higher and most historical cultivars presented lower total chlorophyll levels than did the standard cv. ‘Bavaria’. With respect to the total Al content in the root cells, only cv. ‘Kirsna DS’ was observed to accumulate significantly less Al than the standard cultivar ‘Bavaria’. In addition, the measured parameters exhibited unusual correlations, such as a statistically significant negative correlation between the MDA content in the roots and root cell viability, as well as the absolute root length and root response in the 2 mM Al environment (Table S4). For these reasons, the results of the observations of individual biochemical parameters can be found in Tables S5–S7.

All the biochemical assay data were incorporated into summary PCAs, together with the genetic and morphological data.

### 2.3. Investigation of Al-Resistance-Associated Genotypes in Lithuanian Barley Cultivars

A total of three polymorphisms previously associated with resistance to Al toxicity [17,18,19] were investigated in this study. These polymorphisms included two indel polymorphisms located in the untranslated regions of the gene—one in the 5′ UTR and another in the 3′ UTR—and a single nucleotide polymorphism (SNP) at position 1198. The 5′ UTR polymorphism is characterized by a 1 kb insert, and its presence is indicative of Al resistance. In contrast, the 3′ UTR features a 21 bp indel variant, and the absence of this variant is associated with Al resistance. The SNP at position 1198 within the open reading frame (ORF) involves a transition between T and G nucleotides, with G being linked to resistance and T to susceptibility.

Interestingly, none of the tested cultivars, neither modern nor historical, presented the 5′ UTR polymorphism, which would have been detectable by the formation of an 1844 bp fragment. However, among the modern cultivars, two—the standard resistant cv. ‘Bavaria’ and the Lithuanian cv. ‘Noja DS’—possessed the Al-resistance-associated 3′ UTR deletion. Both of these cultivars also presented the G nucleotide at position 1198 in the ORF.

**Figure 3:**
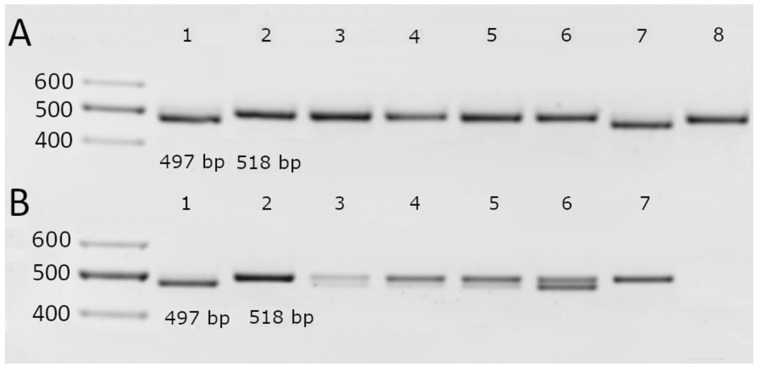
Identification of a 21 bp indel polymorphism in the 3′ UTR of the *HvAACT1* gene in the modern (**A**) and historical (**B**) barley cultivars. A, 1–8—‘Bavaria’, ‘Morex’, ‘Alisa DS’, ‘Arka DS’, ‘Ema DS’, ‘Kirsna DS’, ‘Noja DS’, and ‘Rusnė DS’. B, 1–7—‘Aidas’, ‘Alsa’, Auksiniai II’, ‘Auksiniai 3’, ‘Džiugiai’, ‘Luokė’, and ‘Ūla’.

Among the historical cultivars, cv. ‘Aidas’ presented the same genotype as cv. ‘Bavaria’ and cv. ‘Noja DS’, with both the 3′ UTR deletion and a G at position 1198 (Figure 3, Table 3). The other cultivars were intracultivar heterogeneous for both molecular markers, with the exception of cv. ‘Ūla’, which, while heterogeneous at the position 1198 SNP, did not exhibit the 3′ UTR deletion, whereas cv. ‘Alsa’ had no deletion in the 3′ UTR and a T at position 1198 (Figure 3). A summary of the results is provided in Table 3.

### 2.4. Multivariate Analyses

**Figure 4:**
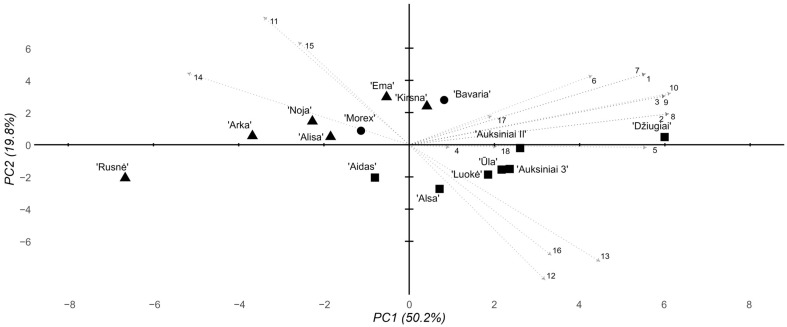
Summary PCA of the morphological, biochemical, and genotyping parameters of the tested barley cultivars under 2 mM Al stress conditions. Eigenvectors are marked according to the following parameters 1–18: 1–3, root response, days 1–3; 4–6, shoot response, days 1–3; 7–9, RTI, days 1–3; 10, average 3-day RTI; 11, total chlorophyll content; 12, chlorophyll a/b ratio; 13, chlorophyll/carotenoid ratio; 14, lipid peroxidation level; 15, Al content in the root cells; 16, root cell viability; 17, 3′ UTR deletion; 18, pos. 1198 SNP. The shapes represent cultivar groups, with circles for standard foreign, triangles for modern Lithuanian, and squares for historical Lithuanian.

All the data collected from the morphometric, biochemical, and genetic *HvAACT1* polymorphism assays were combined and subjected to multidimensional analyses to uncover trends across all the completed investigations. A principal component analysis (PCA) was employed to achieve this goal. Two multivariate analyses were performed, one with all the data and biochemical assays of the plant groups in the 2 mM Al stress environments and the other with all the biochemical data for the 8 mM Al-environment-treated plants. Considering the 2 mM Al stress environment, the first PCA component accounted for a substantial portion of the variability at 50.2% (Figure 4). However, other components did not capture as much variability, with the second representing 19.8%. The cultivars formed two distinct clusters, with the modern and historical Lithuanian cultivars separated across both components. In the modern cultivar cluster, two subclusters were formed, one with cv. ‘Ema DS’, cv. ‘Kirsna DS’, and cv. ‘Bavaria’—the standard Al-resistant foreign cultivar and the two most promising Lithuanian candidates. The other cluster included cv. ‘Morex’ and the remaining modern Lithuanian cultivars. The historical cultivar cluster was more uniform, except for cv. ‘Džiugiai’, which seems to have formed a cluster away from both the modern and historical cultivars.

**Figure 5:**
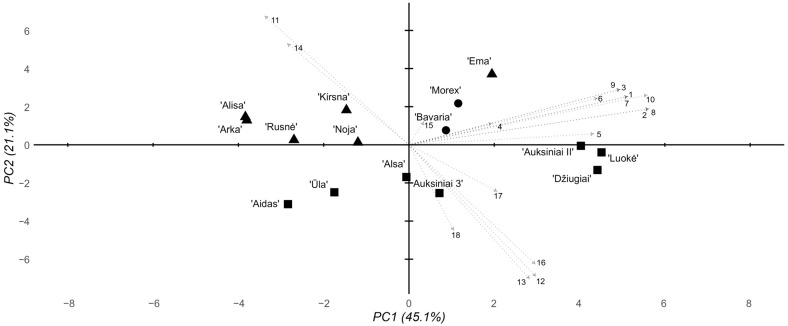
Summary PCA of the morphological, biochemical, and genotyping parameters of the tested barley cultivars under 8 mM stress conditions. Eigenvectors are marked according to the following parameters 1–18: 1–3, root response, days 1–3; 4–6, shoot response, days 1–3; 7–9, RTI, days 1–3; 10, average 3-day RTI; 11, total chlorophyll content; 12, chlorophyll a/b ratio; 13, chlorophyll/carotenoid ratio; 14, lipid peroxidation level; 15, Al content in the root cells; 16, root cell viability; 17, 3′ UTR deletion; 18, pos. 1198 SNP. The shapes represent cultivar groups, with circles for standard foreign, triangles for modern Lithuanian, and squares for historical Lithuanian.

The multidimensional analysis of the morphological and biochemical data from the 8 mM Al stress groups revealed main trends similar to those of the 2 mM stress groups (Figure 5). PC1 still accounted for the majority of the variability at 45.1%, where PC2 accounted for 21.1%. As before, two main historical and modern cultivar clusters were observed. At 8 mM Al, however, the subclusters observed in the 2 mM Al data were shifted—instead of a cv. ‘Ema DS’, cv. ‘Kirsna DS’, and cv. ‘Bavaria’ cluster, cv. ‘Bavaria’, cv. ‘Morex’, and cv. ‘Ema DS’ clustered together, where other cultivars made up another subcluster, highlighting the loss of Al poisoning resistance in cv. ‘Bavaria’ at relatively high Al concentrations. The historical cultivar cluster also shifted, where cv. ‘Džiugiai’ formed a distinct subcluster with cv. ‘Auksiniai II’ and cv. ‘Luokė’.

### 2.5. Expression Levels of Organic Acid Transporters and Aquaporins in Barley Roots

To investigate the discrepancy between the empirical findings, which suggest that cv. ‘Ema DS’ and cv. ‘Kirsna DS’ are potentially the most Al-resistant cultivars assayed, and the genetic evidence indicating that cv. ‘Noja DS’ is the only Lithuanian cultivar positive for both Al-resistance-associated polymorphisms, we conducted gene expression assays for the Al-activated citrate transporter (*HvAACT1*) and the Al-activated malate transporter (*HvALMT1*). *HvAACT1* was previously identified as the primary driver of the Al-poisoning-resistant phenotype in barley, whereas *HvALMT1* has been found to play a similar role in wheat. We selected cultivars ‘Bavaria’, ‘Morex’, ‘Noja DS’, and ‘Ema DS’ for an analysis of the transmembrane transporter gene activity, as they include two foreign cultivars with known Al resistance phenotypes and two Lithuanian cultivars, one with an Al-resistance-associated genotype and the other with an Al-resistance-associated phenotype.

**Figure 6:**
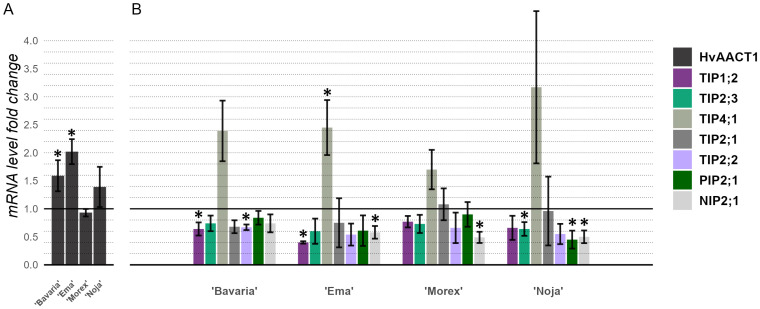
Changes in the levels of expression of *HvAACT1* (**A**) and seven aquaporin genes (**B**) in the roots of the four assayed cultivars after 72 h of 2 mM Al stress. The *HvAACT1* expression change significance comparisons were performed with the Al-susceptible cv. ‘Morex’, while the aquaporin expression change significance comparisons were performed with the base untreated control level expression via a Kruskal–Wallis test with Dunn’s post hoc test for pairwise comparisons, *n* = 3. * *p*  <  0.05.

The *HvALMT1* expression levels were determined to be too low for any meaningful interpretation. As expected, *HvAACT1* was expressed at a reasonable level. As depicted in Figure 6A, 72 h of 2 mM Al stress increased the expression levels of *HvAACT1* in all the analyzed cultivars, except for cv. ‘Morex’, where the expression levels decreased insignificantly. The cultivar ‘Ema DS’ presented the highest relative expression level of *HvAACT1*, with a 2.02-fold increase, followed by cv. ‘Bavaria’, with a 1.59-fold increase, and cv. ‘Noja DS’, with a 1.39-fold increase. Interestingly, this trend suggests the presence of an unknown genetic polymorphism in cv. ‘Ema DS’ that drove the increased expression of *HvAACT1* compared with those in cv. ‘Noja DS’ and cv. ‘Bavaria’, both of which possess the previously identified Al-resistant genotype.

To examine the possible causes of Al resistance in specific cultivars, the expression levels of several aquaporin membrane transporter genes, some of which have been implicated in Al tolerance systems, were measured (Figure 6B). Given that the stress conditions were the same as those used for the *HvAACT1* and *HvALMT1* gene expression analyses, the majority of the tested aquaporins did not exhibit changes in their expression patterns between the stressed and control environments. One exception was the *HvTIP4;1* gene, which displayed a major increase in expression in all the analyzed cultivars, and notably, the largest increase was observed in cv. ‘Ema DS’, which was identified in previous assays as the most promising cultivar in terms of Al resistance.

### 2.6. Sequence Analysis of the HvAACT1 Gene and 1 kb Upstream Region

To determine the cause of the increased *HvAACT1* expression in cv. ‘Ema DS’, we sequenced the *HvAACT1* gene and a 1 kb upstream region. The sequence analysis involved three cultivars, ‘Bavaria’, ‘Noja DS’, and ‘Ema DS’, and excluded cv. ‘Morex’ because of its already available genome assembly (MorexV3_pseudomolecules_assembly, INSDC Assembly GCA_904849725.1, April 2021). Our findings revealed four positional variants in the *HvAACT1* gene. One was previously examined during the genotyping of variants associated with increased Al resistance. At position -−169, we identified an unannotated C>G variant, with cv. ‘Morex’ and cv. ‘Ema DS’ displaying a C and ‘Bavaria’ and ‘Noja DS’ a G. Its significance remains uncertain. We re-confirmed the variant at position 1198, where cv. ‘Bavaria’ and cv. ‘Noja DS’ had a G and cv. ‘Morex’ and cv. ‘Ema DS’ had a T. A known synonymous variant (G>A) was found at position 2283, where cv. ‘Morex’ and cv. ‘Ema DS’ exhibited a G, and cv. ‘Bavaria’ and cv. ‘Noja DS’ an A. Lastly, a recognized TA indel was detected at positions 3644–3645, which were present in cv. ‘Noja DS’ and cv. ‘Ema DS’. The summarized sequencing data are presented in Table 4.

### 2.7. Sequence Analysis of the HvALMT1 Gene

Despite the lack of *HvALMT1* expression in the barley seedling roots, further genotyping was performed to rule out the possibility that the *HvALMT1* genotype interacted with the Al resistance phenotype. The *HvALMT1* gene of all assayed cultivars, excluding cv. ‘Morex’, was sequenced. We observed two positional variants in the *HvALMT1* sequence, both of which were not annotated. Interestingly, all the assayed cultivars carried the same nucleotide at both positions—A at position 1471 and G at position 2254. Conversely, cv. ‘Morex’ had a G at position 1471 and an A at position 2254. The significance of these polymorphisms is unclear because Lithuanian barley cultivars carrying the same genotype vary widely in terms of their Al resistance phenotype. The summarized data are presented in Table 5.

## 3. Discussion

Since the beginning of barley breeding in 1924 at the Dotnuva Plant Breeding Station (Akademija, Lithuania), twenty-eight spring barley cultivars have been created in Lithuania, fifteen of which have been registered on the national and/or European List of Plant Varieties [27]. The present study aimed to assess the Al resistance of six modern and seven historical Lithuanian spring barley cultivars representing the full period of barley breeding history in Lithuania and to re-evaluate the applicability and congruence between the well-established biomarkers associated with Al resistance in barley. Among the tested genotypes, at least two historical cultivars could be considered Al resistant, namely, cv. ‘Džiugiai’, which was selected from local landraces of the Simnas surroundings in 1947 and is characterized by relatively high yields in strongly or moderately acidic (pH 4.5–6.0) soil [28,29], and cv. ‘Auksiniai II’, which was created in 1947, the pedigree of which includes the Al-resistant cv. ‘Bavaria’ (Figure S1), which was used as an Al-resistant genotype in the present study. Our study consisted of four interconnected layers: (I) barley cultivars with the most and least pronounced Al resistance were selected by measuring the absolute and relative morphometric parameters (root and shoot lengths), which reflected the overall biological response to Al stress regardless of the mechanism; (II) widely exploited biochemical markers of the Al stress response, including the shoot pigment content, MDA concentration in the roots and leaves, cell viability, and Al uptake of roots, which were assessed in parallel with the morphometric parameters to screen the most reliable combination for screening of Al-resistant genotypes; (III) sequence and expression analyses of citrate and malate transporter genes in the selected barley cultivars that contrasted most in sensitivity to Al; and (IV) Al-induced changes in the expression of seven aquaporin genes representing the major intrinsic proteins localized in different cell compartments, which were not previously tested in barley in the context of Al-stress conditions.

The root-length-based absolute and relative morphometric parameters appeared to be much more consistent over the time scale than the shoots (Table 1 and Table 2). In this respect, both historical Lithuanian cultivars with expected Al resistance, cvs ‘Džiugiai’ and ‘Auksiniai II’, and a relatively newly created cv. ‘Ema DS’ were the most Al-resistant among all the tested genotypes under both strengths of the Al background (Figure 2). Interestingly, the standard cultivars ‘Bavaria’ (Al-resistant) and ‘Morex’ (Al-sensitive) grouped closely in the central part of the PCA space and did not dramatically differ from one another; hence, they can both be considered only moderately resistant/sensitive to Al stress, at least in the context of Lithuanian barley cultivars. The more pronounced effect of Al, especially at a higher dose, on root growth than on shoot growth is associated with the same Al-induced effect in other cereals, such as rice [30,31,32], as well as in some woody plant species [33,34]; this phenomenon in Al non-accumulators can be at least partially explained by Al accumulation in root tissues due to its low mobility [35]. In addition to low mobility, root tissue is particularly sensitive to Al poisoning compared with more mature tissues of seedling shoots due to the rapidly expanding nature of the apical meristem and the elongation zone of roots, resulting in greater disruption to the cellular and tissue growth processes via the effect of Al itself, or byproducts of Al poisoning, such as enhanced oxidative stress [36,37].

In contrast to the morphometry, the biochemical parameters measured after the exposure of various barley genotypes to the Al stress were controversial (Tables S5–S7), e.g., the MDA content in the roots after the 2 mM Al treatment was significantly negatively correlated with the root cell viability after the 2 mM and 8 mM Al treatments (*p* = 0.02 and *p* = 0.006, respectively; Table S4), which contrasts with the findings of most other studies, which indicate that both parameters tend to be positively correlated with the response of plants to various abiotic stresses [38,39]. Furthermore, despite the inclusion of additional experimental replicates (for biochemical analysis, 5–7 independent runs were performed in total), most of the Al-induced shifts in the studied biochemical parameters were either insignificant or inconsistent, thus making no appreciable contribution to the discrimination between the Al-resistant and Al-susceptible genotypes (cf. Figure 2, Figure 4 and Figure 5). The poor repeatability of the biochemical data may be related to slight fluctuations in the experimental parameters, such as the temperature and pH, which influenced the effective Al concentration in the nutrient solution [2]. Similar scarce correspondence between the widely used biochemical markers in response to Al stress and their strong dependency on genotype and even experimental condition has also been demonstrated in several other plant species [40,41,42,43]. However, the lack of significance in the biochemical response to Al stress may be related to the nature of the colorimetric analysis. The specificity problem of specific colorimetric assays is a well-known problem, especially considering the lipid peroxidation level determination by the widely used thiobarbituric acid-based reaction, which turns into a colored compound not only after the reaction with MDA but also with a variety of non-target chemical species, including sugars and pigments [44,45], the levels of which are not necessarily related to the oxidative stress levels, thus leading to an overestimation of the lipid peroxidation level. In general, our data show highly conflicting results between the root morphometric analysis, which is widely accepted as the most sensitive parameter of plant sensitivity to Al stress [32,46,47], and the most popular colorimetry-based biochemical test. A similar generalization was made in the study by Awasthi et al. (2017) [48], which showed morphometric parameters (relative root reduction and absolute root length) to be the most valuable for the screening of the most Al-tolerant and Al-sensitive rice varieties.

Together with the morphometric and biochemical markers, all the tested barley genotypes were subjected to the analysis of three well-established genetic markers in the barley citrate transporter *HvAACT1* (*HvMATE*), which is considered the key player in barley Al resistance: a 1 kb insert in the 5′ UTR, a 21 bp deletion in the 3′ UTR, and an SNP at position 1198 in the coding region. None of the tested barley genotypes possessed the 1 kb insertion in the 5′ UTR of *HvAACT1*, which is consistent with the findings of the study by Fujii et al. (2012) [17], who reported the insertion in only 20 (out of 265 tested) barley accessions grown in acidic soil areas in the Far East region. However, according to the 21 bp deletion in the 3′ UTR and the SNP at position 1198 (both genetic markers are highly correlated [19]), all the tested barley genotypes fell into three groups: cultivars with or without the deletion and heterogeneous cultivars consisting of individuals with different statuses for the 21 bp deletion (Figure 3 and Table 3). The latter group primarily comprised historical cultivars, including ‘Auksiniai II’ and ‘Džiugiai’, which were included in this study for their potential Al tolerance. Observing this genetic heterogeneity in historical cultivars addresses the global establishment of more rigorous requirements for intracultivar uniformity for newly created cultivars using the DUS system developed in 1961 [26] to significantly contribute to the dramatic genetic erosion of most modern edible cultivars, including cereals [49,50,51]. Owing to the intracultivar heterogeneity of the historical cultivars, the RTI of the historical cv. ‘Džiugiai’ presented the highest Al resistance among the tested cultivars only for lower (2 mM) but not elevated (8 mM) Al concentrations, whereas the RTI of cv. ‘Auksiniai II’ at both Al concentrations was appreciably high but not exceptional among all the tested cultivars (Figure 1 and Figure 2). Considering the abovementioned intracultivar heterogeneity phenomena regarding the Al resistance genetic marker *HvAACT1*, the extents of the Al resistance and some quantitative traits that are undesirable and incompatible with modern agrotechnology, including straw height [52], both potentially Al-resistant historical barley genotypes cannot be considered reliable Al resistance gene donors for the creation of modern cultivars and, consequently, together with other historical genotypes, they were excluded from further analysis.

In general, the genetic analysis revealed the presence of Al-resistance-related genetic markers in the *HvAACT1* gene from the Al-resistant standard genotype ‘Bavaria’ and two Lithuanian barley cultivars, the modern cv. ‘Noja DS’ and the historical cv. ‘Aidas’ (Table 3), both of which expressed only moderate or even weak Al resistance according to the RTI (Figure 1 and Figure 2, respectively). To reveal the possible causes of the discrepancy between the genetic markers in *HvAACT1* and empirical Al resistance, an *HvAACT1* expression analysis was performed in the set of the following selected barley genotypes: the Al-resistant and Al-sensitive standard genotypes (cv. ‘Bavaria’ and cv. ‘Morex’, respectively); the modern cv. ‘Noja DS’, which had the same Al resistance genetic markers as cv. ‘Bavaria’ in *HvAACT1* but was only moderately Al-resistant; and modern cv. ‘Ema DS’, which had the best empirical performance under Al stress but no Al resistance genetic markers in *HvAACT1*. As expected, cvs ‘Bavaria’ and ‘Noja DS’, which possessed the same genetic markers in *HvAACT1*, presented the same level of *HvAACT1* expression in the roots, which was significantly greater than that of the Al-sensitive cv. ‘Morex’. Surprisingly, the highest expression of *HvAACT1* was detected in cv. ‘Ema DS’, which possessed none of the known Al-resistance-related markers in the *HvAACT1* gene (Figure 6). A sequence analysis of the *HvAACT1* putative core promoter encompassing the 1 kb upstream region revealed four variant positions (Table 4), three of which cv. ‘Ema DS’ shared with the Al-sensitive cv. ‘Morex’ and one with cv. ‘Noja DS’. Since none of the determined variant positions in the putative promoter region of *HvAACT1* were unique to cv. ‘Ema DS’, its high *HvAACT1* expression level may be related either to unknown regulatory elements within the non-coding sequences in the gene body or to enhancers located more distantly than 1 kb upstream of the promoter region of *HvAACT1*. Our results strongly suggest that at least in the case of *HvAACT1*, expression analysis should be preferred over sequence analysis in barley Al resistance screening studies. In parallel to *HvAACT1*, the expression of the barley Al-activated malate transporter *HvALMT1*, whose homologs are key players in Al resistance in many plant species [53], was also determined in the barley roots. Experiments with a limited list of barley genotypes demonstrated that a low level of *HvALMT1* expression occurs throughout the plant body and is more pronounced in the roots than in the shoots, and its intensity is weaker in the Al-resistant cultivar than in the Al-sensitive cultivar [54]. Our study revealed only a negligible level of *HvALMT1* in the roots of all four tested barley genotypes, confirming that the role of this transporter in Al resistance in barley is insignificant.

Despite the prevailing trend in barley Al resistance studies to focus mostly on external Al resistance mechanisms (Al exclusion), the internal mechanisms involved in coping with Al within the plant body deserve more attention since barley Al tolerance is a complex trait that may be quantitatively inherited and is less well characterized [55,56,57]. A part of our study focused on evaluating the Al-induced changes in the expression of some barley aquaporins, which can transport diverse non-aqua substrates, thus alleviating the stress caused by metalloids and (heavy) metals [58,59,60,61], including Al [62,63,64], in a variety of plant species, and were not explored in barley in the context of Al stress. Our analysis revealed that among seven (out of forty known [65]) tested barley aquaporins belonging to different families, only *HvTIP4;1* (Tonoplast Intrinsic Protein4;1) expression in the barley roots was upregulated in response to the Al stress, whereas the other tested aquaporins were downregulated (Figure 6). Ligaba et al. (2011) [66] reported that the expression of *HvTIP4;1* in barley roots is upregulated by abscisic acid, the synthesis of which in roots is known to be rapidly induced in response to Al exposure [34,67,68]; hence, the Al-induced molecular consequences in barley roots are comparable with the effects of some heavy metals, which induce water stress at the molecular level [69]. Interestingly, the Al-induced increase in the *HvTIP4;1* expression described in our study was not observed after treatment with any heavy metal [66], suggesting that *HvTIP4;1* is essential and specific for internal Al tolerance in barley, along with proposed internal Al-tolerance-related barley genes such as *HvABCB25* [70], *HvHOX9* [56], and *HvNIP1;2* [57]. Despite the majority of studies indicating the importance of plant-specific Nodulin 26-like intrinsic protein (NIP) family proteins in the internal detoxification of metalloids and metals, including Al [58,65,71], our empirical evidence of TIP family involvement in the barley Al stress response addresses the limited number of studies demonstrating the participation of TIP in intravacuolar Al sequestration in other species, including *Hydrangea* [62] and *Anthoxanthum* [72]. To our knowledge, the direct involvement of aquaporin family genes in the barley response to Al stress is described here for the first time.

## 4. Materials and Methods

### 4.1. Plant Material

Six modern Lithuanian barley cultivars, ‘Alisa DS’, ‘Arka DS’, ‘Ema DS’, ‘Kirsna DS’, ‘Noja DS’, and ‘Rusnė DS’, as well as seven historical Lithuanian cultivars, ‘Aidas’, ‘Alsa’, ‘Auksiniai II’, ‘Auksiniai 3’, ‘Džiugiai’, ‘Luokė’, and ‘Ūla’, were assayed in this investigation; they were procured from the Lithuanian Agrarian and Forestry Sciences Centre Agriculture Institute collection. Two standard foreign cultivars, cv. ‘Morex’ and cv. ‘Bavaria’, which are susceptible and resistant to Al poisoning, respectively, were obtained from the IPK (Leibniz—Institute of Plant Genetics and Cultivated Plant Research) gene bank in Germany. The pedigrees, creation times, and registrations of the Lithuanian National List of Plant Varieties are presented in Table 6.

### 4.2. Grain Sterilization and Al Stress Induction

The grains were surface sterilized according to Ramakrishna et al. (1991) [73] using commercial bleach by immersing them in 70% ethanol for 1 min and then soaking them in 50% commercial bleach solution for 20 min. After this sterilization, the grains were rinsed 5 times for at least 2 min. The grains were then soaked in deionized water for 12 h for imbibition and placed on damp filter paper for 48 h to germinate.

The Al treatment was performed on a filter paper according to Huttová et al. (2002) [74], with the doses selected according to Tamás et al. (2004) [75]. In brief, the germinated seeds were moved to separate germination trays with filter paper soaked with 0.2 mM CaCl_2_, 2 mM AlCl_3_ with 0.2 mM CaCl_2_, and 8 mM AlCl_3_ with 0.2 mM CaCl_2_ solutions, normalized to pH 4.5. Seedling trays were then placed in growth chambers with a 16/8 h daylight cycle at 22 °C and constant humidity.

The plant material for the biochemical analyses was collected after 72 h of exposure to these stressful environments.

All the analyses were performed with a minimum of three to seven independent biological replicates to ensure accuracy and reliability.

### 4.3. Quantifying the Effects of Al Stress on Barley Seedling Morphology

The morphometric parameters of the barley seedlings were evaluated before the transfer to stressful environments and at 24-, 48-, and 72-h intervals after the transfer. The absolute root length (ABS RL, cm) and shoot length (ABS SL, cm) were measured to assess the impacts of the Al toxicity. These impacts were quantified by calculating two key metrics: the relative root or shoot length reduction (RRLR or RSLR, %) and the root tolerance index (RTI). The calculations were as follows:

(1) $$\;RRLR\;or\;RSLR\left(\%\right)=\;\frac{average\left(stress\;group\;RL\;or\;SL\right)-average\left(control\;group\;RL\;or\;SL\right)}{average\left(control\;group\;RL\;or\;SL\right)}*100$$

(2) $$\;RTI=\frac{average\left(stress\;group\;RL\right)}{average\left(control\;group\;RL\right)}$$

The pooled data of at least 10 individuals per treatment per biological replicate were analyzed for each time point, totaling at least 30 individuals per time point.

### 4.4. Evaluation of Biochemical Markers Using Colorimetric Assays

Measurements of the chlorophyll and carotenoid concentrations (mg/g FW), levels of lipid peroxidation in the root and shoot cells (nmol/g FW), Al uptake in the root cells (OD at 490 nm), and root tip cell viability (OD at 600 nm) (*n* = 3 per treatment per biological replicate for all measurements) were performed according to protocols previously designed by Minocha et al. (2009), Hodges et al. (1999), Pandey et al. (2013) and Tamás et al. (2004) [44,75,76,77], respectively. All biochemical assay samples were processed immediately after harvesting and kept on ice throughout the experiments.

### 4.5. Extraction of Barley Seedling Genomic DNA

Genomic DNA from the barley seedlings five days post-sowing was extracted by following a modified CTAB extraction method described previously by Doyle (1991) [78]. In brief, 80–100 mg samples of shoot material were homogenized in liquid nitrogen, and following this procedure, the extracted DNA was dissolved in 100 μL of sterile deionized water. The concentration and purity of the DNA mixture were determined using a Nanodrop 2000 spectrophotometer (Thermo Scientific, Waltham, MA, USA). All further genetic analyses were performed using pooled equimolar DNA samples extracted from 10 individual plants.

### 4.6. HvAACT1 Sequence Analysis

To identify an SNP at position 1198 of the barley *HvAACT1* gene, specific sequencing primers were designed: fw CAACATCATTCGTCGCTGAAG and rv TCAGTCCTGGGGTGAAATCAA. Following the PCR, target amplicons for sequencing were purified by electrophoretic fractioning and the protocol from the Zymoclean Gel DNA Recover Kit (Zymo Research, Irvine, CA, USA). Sanger sequencing was performed by BaseClear (Leiden, The Netherlands).

To identify the 5′ and 3′ UTR indel polymorphisms in the *HvAACT1* gene, electrophoretic fractioning was applied. For the 3′ UTR 21 bp deletion, the PCR products were fractionated on a 3% agarose gel at 30 V for 24 h. The sequences of primers used here were as follows: for the 5′ UTR insertion, fw GGTCCAACACTCTACCCTTCCTT and rv GGTGCGAGTTGCCCCTAGCTATTACAGA, which amplified 1844 bp and 821 bp fragments with or without the insertion, respectively. The primers used for the detection of the 21 bp deletion in the 3′ UTR were fw GCTAGGGCTTGAAAACTGTTTG and rv GACGAACTGTACGATGATGATGC, which amplified 518 bp and 497 bp fragments without or with the deletion, respectively.

To genotype the *HvAACT1* gene polymorphisms, Sanger sequencing of the full gene sequence was performed using seven primer pairs designed to amplify 700–800 bp overlapping fragments (Table S2).

Amplicons were sequenced from both ends, and the reads were mapped to the cv. ‘Morex’ reference genome assembly MorexV3_pseudomolecules_assembly, INSDC Assembly GCA_904849725.1, Apr 2021. Mapping and variant identification were performed using ‘Unipro UGENE v51.0’ (Novosibirsk, Russia) software.

### 4.7. RNA Extraction and cDNA Synthesis for Gene Expression Analysis

RNA extraction from Al-treated and control barley roots was performed after 72 h of exposure using cumulative root samples obtained from five individual seedlings by combining three 2 cm long root tips from each individual for a total of 60–70 mg of plant material. RNA extraction was performed using the commercial innuPREP Plant RNA Kit (Analytik Jena GmbH, Jena, Germany) according to the manufacturer’s recommendations. The residual DNA from the total RNA samples was eliminated using the innuPREP DNase I Digest Kit (Analytik Jena GmbH, Jena, Germany). Immediately after the RNA extraction, cDNA was synthesized using the High-Capacity cDNA Reverse Transcription Kit with an RNase Inhibitor (Thermo Scientific, Vilnius, Lithuania) according to the manufacturer’s protocol. cDNA samples were stored at −80 °C and utilized without re-freezing. Further gene expression assays were performed using 1:20 diluted cDNA samples.

### 4.8. Quantitative PCR Assays for Gene Expression Changes Under Al Stress

Gene expression changes in barley seedling roots exposed to Al stress were determined using the Maxima SYBR Green/ROX qPCR Master Mix Kit (Thermo Scientific, Vilnius, Lithuania). The ADP-ribosylation factor 1-like protein housekeeping gene (*HvADP*) was used as an endogenous control for comparisons [79]. The sequences of primers used for the qPCR of aquaporin family gene expression were obtained from Ligaba et al. (2011) [66], and some of the sequences were redesigned. The sequences of the qPCR primers used here are listed in Table S3. The SYBR green fluorescence system was employed for the qPCR experiments using a Quant Studio 7 Pro (Applied Biosystems, Singapore, Singapore) PCR machine. The gene expression fold change in the Al stress environment was calculated using the ΔΔ*CT* [80] method.

### 4.9. Statistical Analysis

All the experiments were performed at least in triplicate. The mean values presented in the tables and figures are accompanied by their respective standard errors of means. To evaluate the data normality, both Shapiro–Wilk and Anderson–Darling tests were employed. Because some datasets deviated from a normal distribution, nonparametric ANOVA (Kruskal–Wallis test) was conducted for group comparisons, with a post hoc Dunn test for multiple pairwise comparisons. The principal component analysis (PCA) and figure preparation were performed using the R statistical software package, version 4.4.0.

## 5. Conclusions

Our data confirm that the early assessment of barley Al resistance via morphometric root parameters can be replaced neither by colorimetric assays for biochemical markers nor by known genetic markers in citrate and malate transporter genes. In addition, the measurement of citrate transporter expression in the barley roots was the only tested molecular biomarker that coincided with the morphometric root response to Al stress, indicating that both biomarkers are the most informative combination, at least in barley Al resistance screening experiments. The expression of only aquaporin TIP4;1 was upregulated in all the tested spring barley cultivars, suggesting its possible role in intracellular Al sequestration in barley. Of the assayed cultivars, modern Lithuanian cv. ‘Ema DS’ displayed the best and most consistent characteristics under both the mild and severe Al stress, which improved upon even the standard Al-resistant cv. ‘Bavaria’. The performance of cv. ‘Ema DS’ suggests it as a potential candidate for farmers to grow in areas affected by high soil acidity, as well as a candidate for future Al-resistant barley breeding programs.

## Figures and Tables

**Table 1:** Relative shoot length reduction (RSLR, %) of different barley cultivars at the seedling stage to aluminum stress throughout the 72 h experiment.

Cultivar	2 mM			8 mM		
	24 h	48 h	72 h	24 h	48 h	72 h
‘Bavaria’	8.53 ± 0.23	1.13 ± 0.25	1.19 ± 0.35	2.04 ± 0.23	−6.62 ± 0.25	−6.38 ± 0.35
‘Morex’	8.62 ± 0.27	−6.72 ± 0.33	−8.66 ± 0.35 ^a^	9.76 ± 0.30	1.17 ± 0.35	−0.64 ± 0.39
‘Alisa DS’	−6.47 ± 0.24	−11.93 ± 0.22 ^b^	−9.28 ± 0.23 ^c^	−10.83 ± 0.27	−20.63 ± 0.33 ^c^	−20.49 ± 0.37 ^c^
‘Arka DS’	1.50 ± 0.28	−12.45 ± 0.39	−11.96 ± 0.46 ^a^	−3.54 ± 0.28	−12.95 ± 0.36 ^b^	−10.82 ± 0.47 ^b^
‘Ema DS’	−8.70 ± 0.24	−6.76 ± 0.31 ^a^	−1.80 ± 0.44 ^a^	−2.68 ± 0.24	−6.34 ± 0.31 ^a^	−0.27 ± 0.44
‘Kirsna DS’	0.35 ± 0.28	−2.23 ± 0.36	0.22 ± 0.38	0.97 ± 0.28	−6.65 ± 0.36	−4.17 ± 0.38
‘Noja DS’	−6.68 ± 0.28	−8.58 ± 0.34 ^a^	−0.79 ± 0.38	−11.07 ± 0.28	−9.84 ± 0.34 ^a^	−4.47 ± 0.38 ^a^
‘Rusnė DS’	−16.89 ± 0.22 ^c^	−19.72 ± 0.28 ^c^	−23.81 ± 0.35 ^c^	−12.29 ± 0.18 ^c^	−15.55 ± 0.16 ^c^	−13.88 ± 0.17 ^c^
‘Aidas’	6.58 ± 0.34	2.16 ± 0.32	0.95 ± 0.33	−2.05 ± 0.34	−12.65 ± 0.32	−13.85 ± 0.33 ^a^
‘Alsa’	5.52 ± 0.22	0.00 ± 0.34	−7.97 ± 0.44	15.52 ± 0.31	2.20 ± 0.42	−8.20 ± 0.52
‘Auksiniai II’	−10.94 ± 0.27	−6.97 ± 0.39	−10.85 ± 0.43	−1.50 ± 0.20	−6.40 ± 0.20	−6.25 ± 0.26
‘Auksiniai 3’	0.45 ± 0.16	−2.82 ± 0.25	−2.50 ± 0.34	−13.79 ± 0.16 ^a^	−18.06 ± 0.24 ^c^	−14.25 ± 0.27 ^b^
‘Džiugiai’	−9.82 ± 0.58	17.58 ± 1.17	4.24 ± 1.44	−8.28 ± 0.58	11.42 ± 1.17	8.53 ± 1.44
‘Luokė’	2.91 ± 0.18	4.03 ± 0.31	−5.52 ± 0.43 ^a^	8.89 ± 0.18	1.09 ± 0.31	−2.52 ± 0.43
‘Ūla’	−3.23 ± 0.20	−4.13 ± 0.22	−6.66 ± 0.29	−7.38 ± 0.20	−16.06 ± 0.22 ^b^	−14.66 ± 0.29 ^c^

The values are given with the respective SEMs. Negative values represent a decrease in the shoot length compared with the control, while positive values show an increase. Significance comparisons were performed with the same cultivar control group by comparing the averages of the absolute shoot lengths. ^a^ *p* < 0.05; ^b^ *p* < 0.01; ^c^ *p* < 0.001. The dotted lines separate the two foreign standard cultivars, as well as the modern (middle) and historical (bottom) Lithuanian cultivars.

**Table 2:** The dynamics of the relative root length reduction (RRLR, %) in different barley cultivars at the seedling stage under aluminum stress throughout the duration of the experiment.

Cultivar	2 mM			8 mM		
	24 h	48 h	72 h	24 h	48 h	72 h
‘Bavaria’	0.07 ± 0.15	−11.98 ± 0.19 ^b^	−17.83 ± 0.26 ^c^	−3.47 ± 0.15	−21.01 ± 0.19 ^c^	−27.64 ± 0.26 ^c^
‘Morex’	−2.24 ± 0.15	−17.18 ± 0.19 ^c^	−21.67 ± 0.21 ^c^	−5.69 ± 0.15	−19.79 ± 0.19 ^c^	−23.80 ± 0.21 ^c^
‘Alisa DS’	−6.05 ± 0.13	−16.52 ± 0.14 ^c^	−21.82 ± 0.17 ^c^	−13.65 ± 0.13 ^b^	−29.15 ± 0.14 ^c^	−33.26 ± 0.17 ^c^
‘Arka DS’	−4.98 ± 0.15	−24.15 ± 0.22 ^c^	−28.01 ± 0.26 ^c^	−14.78 ± 0.15 ^a^	−30.83 ± 0.22 ^c^	−36.20 ± 0.26 ^c^
‘Ema DS’	−2.20 ± 0.18	−14.84 ± 0.24 ^c^	−13.81 ± 0.33 ^b^	−4.93 ± 0.18	−14.35 ± 0.24 ^c^	−13.65 ± 0.33 ^c^
‘Kirsna DS’	0.33 ± 0.16	−11.74 ± 0.21 ^b^	−16.92 ± 0.24 ^c^	−11.74 ± 0.16 ^a^	−23.57 ± 0.21 ^c^	−30.56 ± 0.24 ^c^
‘Noja DS’	−7.01 ± 0.18	−22.21 ± 0.22 ^c^	−24.30 ± 0.24 ^c^	−12.79 ± 0.18 ^b^	−23.70 ± 0.22 ^c^	−31.38 ± 0.24 ^c^
‘Rusnė DS’	−17.48 ± 0.15 ^c^	−26.73 ± 0.16 ^c^	−36.62 ± 0.20 ^c^	−18.49 ± 0.15 ^c^	−25.86 ± 0.16 ^c^	−28.89 ± 0.19 ^c^
‘Aidas’	−10.30 ± 0.30	−22.08 ± 0.28 ^c^	−24.35 ± 0.36 ^c^	−19.59 ± 0.30 ^b^	−31.56 ± 0.28 ^c^	−36.73 ± 0.36 ^c^
‘Alsa’	−4.17 ± 0.48	−16.00 ± 0.82	−23.08 ± 1.01	−6.02 ± 0.48	−28.00 ± 0.82	−37.29 ± 0.62
‘Auksiniai II’	1.19 ± 0.48	−6.92 ± 0.45	−15.04 ± 0.48	−1.90 ± 0.48	−9.87 ± 0.45	−18.01 ± 0.24 ^a^
‘Auksiniai 3’	−4.60 ± 0.26	−13.08 ± 0.32	−11.91 ± 0.35	−11.50 ± 0.26 ^a^	−21.76 ± 0.32 ^b^	−23.10 ± 0.37 ^b^
‘Džiugiai’	7.82 ± 0.94	−3.85 ± 1.21	−8.31 ± 1.47	−6.38 ± 0.94	−12.88 ± 1.21	−20.00 ± 1.47
‘Luokė’	−3.89 ± 0.27	−13.23 ± 0.33	−18.17 ± 0.40 ^a^	−1.04 ± 0.27	−11.06 ± 0.33	−18.86 ± 0.33 ^a^
‘Ūla’	−0.38 ± 0.24	−10.65 ± 0.33 ^a^	−16.38 ± 0.43 ^b^	−15.45 ± 0.24	−26.81 ± 0.33 ^c^	−33.75 ± 0.33 ^c^

The values are given with the respective SEMs. Negative values represent a decrease in the root length compared with the control, while positive values show an increase. Significance comparisons were performed with the same cultivar control group by comparing the averages of the absolute root lengths. ^a^ *p* < 0.05; ^b^ *p* < 0.01; ^c^ *p* < 0.001. The dotted lines separate the two foreign standard cultivars, as well as the modern (middle) and historical (bottom) Lithuanian cultivars.

**Table 3:** Summary of *HvAACT1* gene sequence variation in analyzed historical and modern Lithuanian barley cultivars.

Cultivar	1 kb Insertion in 5′UTR	21 bp Deletion in 3′UTR	SNP at 1198 Position
‘Bavaria’	Not present	+	G
‘Morex’		−	T
‘Alisa DS’		−	T
‘Arka DS’		−	T
‘Ema DS’		−	T
‘Kirsna DS’		−	T
‘Noja DS’		+	G
‘Rusnė DS’		−	T
‘Aidas’		+	G
‘Alsa’		−	T
‘Auksiniai II’		+/−	T/G
‘Auksiniai 3’		+/−	T/G
‘Džiugiai’		+/−	T/G
‘Luokė’		+/−	T/G
‘Ūla’		−	T/G

The dotted lines separate the two foreign standard cultivars, as well as the modern (middle) and historical (bottom) Lithuanian cultivars.

**Table 4:** Variant positions identified in the *HvAACT1* gene body and 1 kb upstream region.

Cultivar	Position *			
	−169; 531, 411, 112	1198; 531, 412, 479	2283; 531, 413, 564	3644–3645; 531, 414, 925
‘Morex’	C	T	G	GA
‘Bavaria’	G	G	A	GA
‘Noja DS’	G	G	A	GATA
‘Ema DS’	C	T	G	GATA

* In the sequenced PCR product; position in GCA_904849725.1 Morex genome assembly.

**Table 5:** Variant positions identified in *HvALMT1* gene.

Cultivar	Position *	
	1471; 469, 781, 055	2254; 469, 780, 278
‘Morex’	G	A
‘Bavaria’	A	G
All Lithuanian cultivars	A	G

* In sequenced PCR product; position in GCA_904849725.1 Morex genome assembly.

**Table 6:** Barley cultivars assayed for aluminum resistance in this investigation.

Cultivar Name	Origin	Year of Development	Period of Registration	Status of Al Tolerance
‘Morex’ *	‘CI 15773’	1924		Sensitive
‘Bavaria’ *	Local landraces	1978		Tolerant
‘Alisa DS’	‘Jacinta’ × ‘Vortex’	2008	2011–	Unknown
‘Arka DS’	‘LŽI 7385’ × ‘Madona’	2009	2011–	Unknown
‘Ema DS’	‘Mentor’ × ‘Annabell’	2010	2013–	Unknown
‘Kirsna DS’	‘LŽI 7386’ × ‘Orlik’	2010	2013–	Unknown
‘Noja DS’	‘LŽI 7386’ × ‘Pongo’	2009	2012–	Unknown
‘Rusnė DS’	‘Mentor’ × ‘Annabell’	2013	2016–	Unknown
‘Aidas’	(KM-1192 × ‘Ofir’) × ‘Effendi’	1990	1994–2007	Unknown
‘Alsa’	(‘Mirena’ × ‘Gintariniai’ mutant) × (‘Abava‘ × ‘Emir’)	1993	1996–2007	Unknown
‘Auksiniai II’	‘Abed Kenia’ × ‘Ackermanns Isaria’	1947	1950–1990	Suspected tolerant
‘Auksiniai 3’	‘Carina’ × ‘Tarra 26’	1983	1987–2007	Unknown
‘Džiugiai’	Local landraces	1947	1950–1975	Suspected tolerant
‘Luokė’	[‘Vega’ × (‘Ofir’ × ‘Berenice’)] × ‘Flare’	1999	2001–	Unknown
‘Ūla’	‘Roland’ × Ca 33787	1992	1995–2007	Unknown

* indicates cultivars with known aluminum resistance phenotypes—‘Bavaria’ is resistant and ‘Morex’ is susceptible. The dotted lines separate the two foreign standard cultivars (‘Bavaria’—Germany, ‘Morex’—USA), as well as the modern (middle) and historical (bottom) Lithuanian cultivars.

## Data Availability

Processed data are contained within this article or Supplementary Materials. Full dataset available upon request from the authors.

## Supplementary Materials

The following supporting information can be downloaded from https://www.mdpi.com/article/10.3390/ijms26083803/s1.
