# Supplementary material for: Early Seedling Screening Reveals Unidentified Al Resistance Mechanisms in Lithuanian Barley Cultivars
Source: Int J Mol Sci. 2025 Apr 17;26(8):3803. doi: 10.3390/ijms26083803 (PMC12028139; doi:10.3390/ijms26083803)
Supplement: Supplementary file 1 [file ijms-26-03803-s001.zip › ijms-3568987-supplementary.pdf]

Supplemental Material

Table S1. Assayed barley cultivar root tolerance indices (RTIs) over 72 hours of measurement in 2 mM and 8 mM Al stress environments.

Cultivar	2 mM			8 mM		
	24 h	48 h	72 h	24 h	48 h	72 h
‘Bavaria’	1.00±0.23	0.88±0.25	0.82±0.35	0.97±0.23	0.79±0.25	0.72±0.35
‘Morex’	0.98±0.27	0.83±0.33	0.78±0.35	0.94±0.30	0.80±0.35	0.76±0.39
‘Alisa DS’	0.94±0.24	0.83±0.22	0.78±0.23	0.86±0.27	0.71±0.33	0.67±0.37
‘Arka DS’	0.95±0.28	0.76±0.39	0.72±0.46	0.85±0.28	0.69±0.36	0.64±0.47
‘Ema DS’	0.98±0.24	0.85±0.31	0.86±0.44	0.95±0.24	0.86±0.31	0.86±0.44
‘Kirsna DS’	1.00±0.28	0.88±0.36	0.83±0.38	0.88±0.28	0.76±0.36	0.69±0.38
‘Noja DS’	0.93±0.28	0.78±0.34	0.76±0.38	0.87±0.28	0.76±0.34	0.69±0.38
‘Rusnė DS’	0.83±0.22	0.73±0.28	0.63±0.35	0.82±0.18	0.74±0.16	0.71±0.17
‘Aidas’	0.90±0.34	0.78±0.32	0.76±0.33	0.80±0.34	0.68±0.32	0.63±0.33
‘Alsa’	0.96±0.22	0.84±0.34	0.77±0.44	0.94±0.31	0.72±0.42	0.63±0.52
‘Auksiniai II’	1.01±0.27	0.93±0.39	0.85±0.43	0.98±0.20	0.90±0.20	0.82±0.26
‘Auksiniai 3’	0.95±0.16	0.87±0.25	0.88±0.34	0.88±0.16	0.78±0.24	0.77±0.27
‘Džiugiai’	1.08±0.58	0.96±0.63	0.92±0.78	0.94±0.58	0.87±0.63	0.80±0.78
‘Luokė’	0.96±0.18	0.87±0.31	0.82±0.43	0.99±0.18	0.89±0.31	0.81±0.43
‘Ūla’	1.00±0.20	0.89±0.22	0.84±0.29	0.85±0.20	0.73±0.22	0.66±0.29

Table S2. Characteristics of the primers used to amplify seven fragments of the *HvAACT1* gene sequence.

Primer name	Primer direction	Primer sequence (5’ → 3’)	Length, bp
HvAACT1-1	Forward	TCAAATGATGCAGTATTCCTCGC	23
HvAACT1-1	Reverse	ATCGGCTTTTCTCCCTCGAAA	21
HvAACT1-2	Forward	GCTGGGGGTGGTCGCTT	17
HvAACT1-2	Reverse	ATGTTGGTTGGATGGTCGTGA	21
HvAACT1-3	Forward	GGTGAACTGTACCTTGTCGCA	21
HvAACT1-3	Reverse	TACGCTAACAAGCGGGTAGA	20
HvAACT1-4	Forward	CCGTCCTTCTTTCTCCTGGTG	21
HvAACT1-4	Reverse	CGGAAAACGCCCTGCATTG	19
HvAACT1-5	Forward	CACTATTCCAGGACTCACCG	20
HvAACT1-5	Reverse	CCCAGTTCAGCACACTCTCTA	21
HvAACT1-6	Forward	CCGTTGCTGGACAGGTCAGA	20
HvAACT1-6	Reverse	GTAGATGGTCAATGCGACCCA	21
HvAACT1-7	Forward	TGAAACTTGTCCCGAGACTGA	21
HvAACT1-7	Reverse	GATTTTTACAGAGCACTGGTCC	22

Table S3. qPCR primers for the analysis of gene expression in aluminum stress and control environment assays.

Amplified gene	Primer direction	Primer sequence, 5'→ 3'	Length, nt
Endogenous control, *HvADP*	Forward	GCTCTCCAACAACATTGCCAAC	22
Endogenous control, *HvADP*	Reverse	GAGACATCCAGCATCATTCATTCC	24
Citrate transporter *HvAACT1*	Forward	CGTCTAGGTTCGGTGGAGATA	21
Citrate transporter *HvAACT1*	Reverse	GCTAACAAGCGGGTAGATACAG	22
Malate transporter *HvALMT1*	Forward	GCTGAGCAAAGGCTTGAATAG	21
Malate transporter *HvALMT1*	Reverse	GCCGACGCTAGGAAGAAG	18
Aquaporin family gene *HvTIP1;2*	Forward	ACATCAGCCTCCTCAAGGC	19
Aquaporin family gene *HvTIP1;2*	Reverse	CGATGAAGCCGATGGCGG	18
Aquaporin family gene *HvTIP2;1*	Forward	GGGTGGTAAGAGAGAGTGAAAG	22
Aquaporin family gene *HvTIP2;1*	Reverse	TCGGTGAGTTTGCCATAGG	19
Aquaporin family gene *HvTIP2;2*	Forward	GATGGTGAAGCTTGCGTTTG	20
Aquaporin family gene *HvTIP2;2*	Reverse	GTCAGTTGCCCGTAGGAAAT	20
Aquaporin family gene *HvTIP2;3*	Forward	CTACTGGGTTGCGCAGCTC	19
Aquaporin family gene *HvTIP2;3*	Reverse	GTGCCGAGGGATCCCTTC	18
Aquaporin family gene *HvTIP4;1*	Forward	CACCGACAATAAGGCCGGT	19
Aquaporin family gene *HvTIP4;1*	Reverse	CGGTGCTGTACGTGGTGG	18
Aquaporin family gene *HvNIP2;1*	Forward	GATTCAGGTGCCGTTCTACTG	21
Aquaporin family gene *HvNIP2;1*	Reverse	GAAGGTGACGATGATCTCGATG	22
Aquaporin family gene *HvPIP2;1*	Forward	GCTAGCTTAGCAATGGCCAAGGAC	24
Aquaporin family gene *HvPIP2;1*	Reverse	GTCGGACTGGTGCTTGTACC	20

Table S4. Pearson correlation matrix for the biochemical and genetic parameters of the tested barley cultivars.

	RL control	RL 2mM Al	RL 8mM Al	RR% 2mM Al	RR% 8mM Al	RTI% 2mM Al	RTI% 8mM Al	MDA 2mM Al	MDA 8mM Al	Hematoxylin 2mM Al	Hematoxylin 8mM Al	Evans blue 2mM Al	Evans blue 8mM Al	3'UTR 20 bp del	SNP at 1198 position
RL control	1.0000														
RL 2mM Al	**0.9034**	1.0000													
RL 8mM Al	**0.7922**	**0.8760**	1.0000												
RR% 2mM Al	0.2389	**0.6282**	**0.5443**	1.0000											
RR% 8mM Al	-0.1185	0.1546	0.5002	**0.5703**	1.0000										
RTI% 2mM Al	0.2389	**0.6282**	**0.5443**	**1.0000**	**0.5703**	1.0000									
RTI% 8mM Al	-0.1185	0.1546	0.5002	**0.5703**	**1.0000**	**0.5703**	1.0000								
MDA 2mM Al	-0.3393	**-0.5562**	**-0.5811**	**-0.6164**	-0.3808	**-0.6164**	-0.3808	1.0000							
MDA 8mM Al	-0.2627	-0.2807	-0.3711	-0.1474	-0.1526	-0.1474	-0.1526	**0.7777**	1.0000						
Hematoxylin 2mM Al	-0.2804	-0.3409	-0.0789	-0.2324	0.2968	-0.2324	0.2968	0.4898	0.4630	1.0000					
Hematoxylin 8mM Al	-0.0206	0.1188	0.1206	0.2499	0.1803	0.2499	0.1803	0.0100	0.4778	0.3453	1.0000				
Evans blue 2mM Al	**0.5242**	**0.5643**	**0.5660**	0.2627	0.1191	0.2627	0.1191	**-0.5848**	-0.3296	-0.3847	0.2829	1.0000			
Evans blue 8mM Al	**0.5903**	**0.6885**	**0.6791**	0.4288	0.2159	0.4288	0.2159	**-0.6737**	-0.4356	-0.3922	0.2443	**0.9398**	1.0000		
3'UTR del	0.4881	0.4857	**0.5333**	0.2334	0.1682	0.2334	0.1682	-0.2265	-0.2340	0.3065	0.1025	0.0983	0.3146	1.0000	
SNP at 1198 position	**0.7574**	**0.6836**	**0.6241**	0.2030	-0.0268	0.2030	-0.0268	-0.2014	-0.2061	0.0774	0.0338	0.2745	0.4488	0.8981	1.0000

Parameter abbreviations: RL – absolute root length; RR% - root response, %; RTI – root tolerance index; MDA – lipid peroxidation level; Hematoxylin – Al content in root cells; Evans blue – root cell viability. Statistically significant ($P < 0.05$) correlation coefficients are presented in bold.

Table S5. MDA content (nmol/g fw) in barley seedling roots and shoots after 72 h under Al stress and in the control environment.

Cultivar	MDA concentration in shoots			MDA concentration in roots		
	Control	2 mM Al	8 mM Al	Control	2 mM Al	8 mM Al
‘Bavaria’	18.89±1.94	19.68±1.87	19.98±1.41	21.40±3.73	25.31±5.17	24.71±3.81
‘Morex’	22.51±2.26	29.36±3.19[a]	28.97±3.37[a]	21.26±2.40	23.69±4.17	21.37±2.40
‘Alisa DS’	23.13±1.12	23.00±2.00	24.39±3.23	22.30±2.87	25.94±4.30	31.93±6.26
‘Arka DS’	25.23±3.17	23.87±1.66	25.54±2.18	24.99±3.00	27.33±3.31	25.88±3.58
‘Ema DS’	26.04±3.55	27.45±2.76	29.67±5.21	23.51±2.53	24.42±2.85	24.56±3.44
‘Kirsna DS’	22.58±1.71	21.75±3.43	23.48±2.02	21.50±2.61	23.26±3.28	24.05±2.97
‘Noja DS’	24.98±2.95	24.37±1.89	24.62±4.14	22.15±4.23	23.53±3.43	20.69±3.72
‘Rusnė DS’	25.57±2.54	23.45±2.96	26.54±2.62	21.39±3.32	25.97±4.98	20.27±3.38
‘Aidas’				15.54±3.69	23.08±9.29	18.27±3.53
‘Alsa’				16.83±6.89	18.11±5.93	11.06±0.80
‘Auksiniai II’				22.44±7.69	16.83±6.89	18.11±5.93
‘Auksiniai 3’		*ND*		16.51±2.08	17.47±0.16	12.98±0.16
‘Džiugiai’				18.75±6.57	17.47±5.61	17.95±3.53
‘Luokė’				20.19±2.24	19.07±5.61	16.03±3.53
‘Ūla’				18.43±5.61	21.63±5.61	23.88±6.25

Significance comparisons performed with respective ‘Bavaria’ stress group: [a] $P < 0.05$; [b] $P < 0.01$; [c] $P < 0.001$. Dotted lines separate two foreign standard cultivars as well as modern (middle) and historical (bottom) Lithuanian cultivars. ND – no data.

Table S6. Total chlorophyll content and chlorophyll a/b ratio in barley seedling leaves after 72 hours of Al stress.

Cultivar	Total chlorophyll content, mg/g fw			Chlorophyll a/b ratio		
	Control	2 mM Al	8 mM Al	Control	2 mM Al	8 mM Al
‘Bavaria’	1.51±0.04	1.56±0.04	1.31±0.05	2.93±0.09	2.98±0.15	3.19±0.11
‘Morex’	1.42±0.06	1.39±0.03[a]	1.38±0.04	3.25±0.13	3.31±0.09	3.28±0.09
‘Alisa DS’	1.67±0.06	1.39±0.10	1.56±0.05C	2.85±0.09	3.09±0.18	3.03±0.11
‘Arka DS’	1.64±0.03[a]	1.56±0.07	1.55±0.04C	2.89±0.14	3.05±0.11	3.02±0.11
‘Ema DS’	1.56±0.08	1.51±0.16	1.47±0.07C	2.87±0.11	2.16±0.39[a]	3.05±0.11
‘Kirsna DS’	1.50±0.04	1.47±0.08	1.43±0.08	3.06±0.11	3.00±0.14	3.20±0.15
‘Noja DS’	1.44±0.05	1.46±0.05	1.43±0.02	3.11±0.10	2.83±0.11	3.24±0.12
‘Rusnė DS’	1.59±0.03	1.37±0.07[a]	1.38±0.05	2.80±0.12	3.31±0.11	3.30±0.12
‘Aidas’	1.17±0.10[b]	1.17±0.09[b]	1.10±0.04[b]	3.84±0.25[b]	3.67±0.11[b]	3.42±0.09
‘Alsa’	1.28±0.14	1.01±0.12[b]	0.85±0.09[b]	4.13±0.58[a]	3.87±0.10[a]	3.52±0.05[a]
‘Auksiniai II’	1.34±0.06[a]	1.07±0.05	0.97±0.08[b]	3.82±0.19[b]	3.77±0.06[b]	3.66±0.07[b]
‘Auksiniai 3’	1.09±0.07[c]	1.07±0.05C	0.74±0.06[c]	4.02±0.06[c]	3.89±0.09[c]	3.77±0.04[c]
‘Džiugiai’	1.00±0.21[a]	1.02±0.11[b]	0.69±0.10[c]	3.95±0.05[b]	3.93±0.17[a]	3.72±0.02[a]
‘Luokė’	1.40±0.10	1.03±0.06[c]	1.05±0.08[a]	4.05±0.04[c]	3.93±0.09[c]	3.54±0.12[a]
‘Ūla’	1.27±0.05	1.18±0.08[b]	1.03±0.07[c]	3.38±0.42	3.79±0.10[b]	3.74±0.07[b]

Significance comparisons with respective ‘Bavaria’ stress groups: [a]$P < 0.05$; [b]$P < 0.01$; [c]$P < 0.001$. Dotted lines separate two foreign standard cultivars as well as modern (middle) and historical (bottom) Lithuanian cultivars.

Table S7. The aluminum content in seedling root cells, expressed as hematoxylin absorption measured at 490 nm, and the viability of seedling root cells expressed as Evans blue absorption measured at 600 nm, both of which were evaluated after 72 hours in the stressed and control environments.

Cultivar	Aluminum content			Cell viability		
	Control	2 mM Al	8 mM Al	Control	2 mM Al	8 mM Al
‘Bavaria’	0.04±0.02	0.25±0.03	0.47±0.06	0.24±0.02	0.45±0.06	1.21±0.21
‘Morex’	0.03±0.01	0.23±0.02	0.49±0.06	0.17±0.02	0.43±0.02	0.98±0.15
‘Alisa DS’	0.05±0.03	0.22±0.04	0.59±0.07[a]	0.24±0.03	0.54±0.08	1.08±0.27
‘Arka DS’	0.03±0.01	0.17±0.04	0.43±0.05	0.19±0.01	0.35±0.05	0.72±0.14
‘Ema DS’	0.03±0.02	0.21±0.05	0.40±0.07	0.16±0.02	0.33±0.03	0.68±0.14
‘Kirsna DS’	0.03±0.01	0.21±0.04	0.40±0.05[a]	0.18±0.02	0.31±0.08	0.56±0.08
‘Noja DS’	0.04±0.02	0.23±0.04	0.47±0.05	0.18±0.01	0.33±0.07	0.80±0.07
‘Rusnė DS’	0.05±0.02	0.24±0.02	0.43±0.06	0.22±0.01	0.49±0.06	0.99±0.09
‘Aidas’	0.01±0.00	0.17±0.05	0.37±0.07	0.18±0.02	0.57±0.04	1.36±0.13
‘Alsa’	0.01±0.00	0.11±0.02	0.35±0.03	0.12±0.01	0.49±0.06	1.12±0.24
‘Auksiniai II’	0.01±0.00	0.21±0.04	0.54±0.03	0.13±0.02	0.62±0.07	1.45±0.34
‘Auksiniai 3’	0.01±0.00	0.14±0.04	0.46±0.10	0.12±0.01	0.52±0.11	1.41±0.24
‘Džiugiai’	0.02±0.01	0.18±0.01	0.52±0.07	0.19±0.03	0.60±0.07	1.73±0.36
‘Luokė’	0.02±0.00	0.18±0.05	0.39±0.10	0.21±0.05	0.62±0.07	1.62±0.38
‘Ūla’	0.02±0.01	0.10±0.01	0.49±0.17	0.21±0.00	0.64±0.14	1.60±0.30

Significance comparisons with respective control group: [a] $P < 0.05$. Dotted lines separate two foreign standard cultivars as well as modern (middle) and historical (bottom) Lithuanian cultivars.

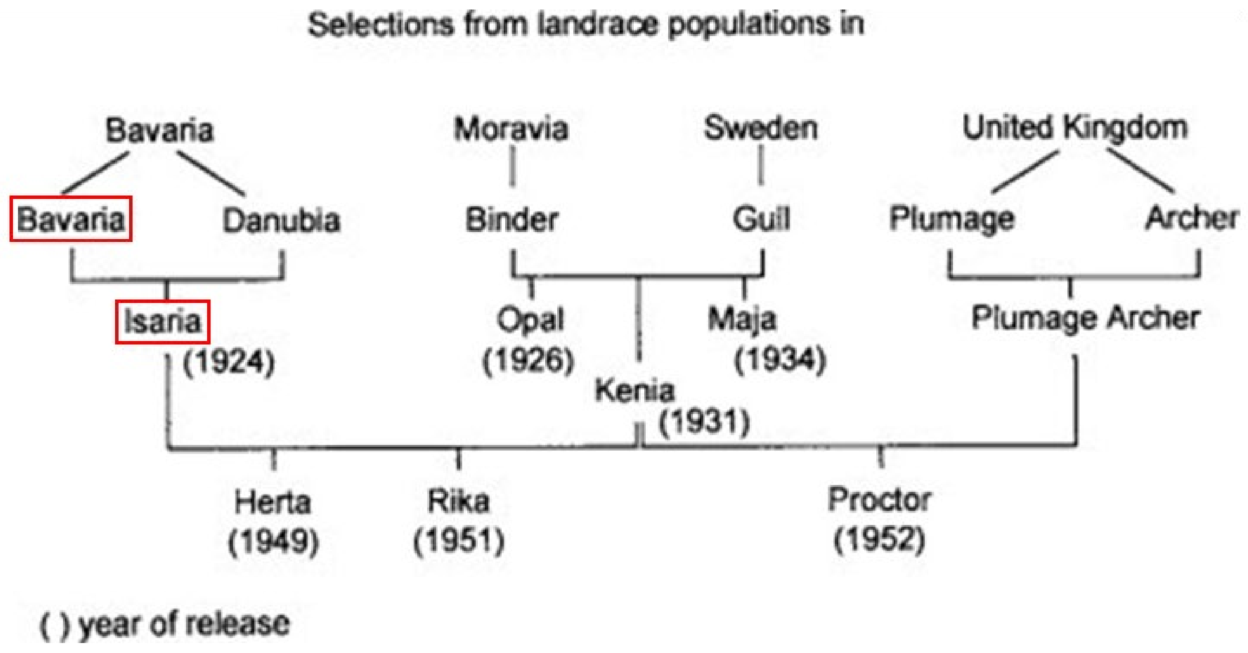

Figure S1. Pedigree of spring barley cultivars derived from early cycles of cross-breeding. Red squares denote two cultivars of interest—Al-resistant cv. 'Bavaria', used in this study as the Al resistance standard, and cv. 'Ackermanns Isaria', from which historical Lithuanian cv. 'Auksiniai II' was derived (modified according to Fishbeck, 2003).
